# Lung lesion detectability on images obtained from decimated and CNN-based denoised [^18^F]-FDG PET/CT scan: an observer-based study for lung-cancer screening

**DOI:** 10.1007/s00259-025-07259-2

**Published:** 2025-04-25

**Authors:** Daphné Faist, Silvano Gnesin, Siria Medici, Alysée Khan, Marie Nicod Lalonde, Niklaus Schaefer, Adrien Depeursinge, Maurizio Conti, Joshua Schaefferkoetter, John O. Prior, Mario Jreige

**Affiliations:** 1 Department of Nuclear Medicine and Molecular Imaging, Lausanne University Hospital and University of Lausanne, Rue du Bugnon 21, CH- 1011 Lausanne, Switzerland; 2 Institute of Radiation Physics, Lausanne University Hospital and University of Lausanne, Rue du Bugnon 21, CH- 1011 Lausanne, Switzerland; 3 Institute of Informatics, School of Management, HES-SO Valais-Wallis University of Applied Sciences and Arts Western Switzerland, Rue du Technopôle 3, CH-3960 Sierre, Switzerland; 4 Siemens Medical Solutions USA, Inc. 810 Innovation Drive, Knoxville, TN 37932 USA

**Keywords:** Low-dose PET, Denoising, Detectability, Lung cancer screening

## Abstract

**Purpose:**

To assess feasibility of lung cancer screening, we analysed lung lesion detectability simulating low-dose and convolutional neural network (CNN) denoised [^18^F]-FDG PET/CT reconstructions.

**Methods:**

Retrospectively, we analysed lung lesions on full statistics and decimated [^18^F]-FDG PET/CT. Reduced count PET data were emulated according to various percentage levels of total. Full and reduced statistics datasets were denoised using a CNN algorithm trained to recreate full statistics PET. Two readers assessed a detectability score from 3 to 0 for each lesion. The resulting detectability score and quantitative measurements were compared between full statistics and the different decimation levels (100%, 30%, 5%, 2%, 1%) with and without denoising.

**Results:**

We analysed 141 lung lesions from 49 patients across 588 reconstructions. The dichotomised lung lesion malignancy score was significantly different from 10% decimation without denoising (*p* < 0.029) and from 5% decimation with denoising (*p* < 0.001). Compared to full statistics, detectability score distribution differed significantly from 2% decimation without denoising (*p* < 0.001) and from 5% decimation with denoising (*p* < 0.001). Detectability scores at same decimation levels with or without denoising differed significantly at 10%, 2%, and 1% decimation (*p* < 0.019); dichotomised scores did not differ significantly. Denoising significantly increased the proportion of lung lesion scores with a high diagnostic confidence (3 and 0) (*p* < 0.038).

**Conclusion:**

Lung lesion detectability was preserved down to 30% of injected activity without denoising and to 10% with denoising. These results support the feasibility of reduced-activity [^18^F]-FDG PET/CT as a potential tool for lung lesion detection. Further studies are warranted to compare this approach with low-dose CT in screening settings.

**Supplementary Information:**

The online version contains supplementary material available at 10.1007/s00259-025-07259-2.

## Introduction

Lung cancer is the leading cause of death in oncological patients with estimated 380′000 death cases in Europe during the year 2020 [1]. In addition to eliminating the main risk factor (tobacco use), early diagnosis of lung cancer is proven essential to reduce mortality through a prompt treatment assessment [2]. Lung cancer screening using low-dose computed tomography (LDCT) has been initiated for high-risk patients, as randomised trials have shown a decrease in mortality and shorter diagnostic delay [3, 4, 5]. Screening is challenging because low-dose CT is sensitive to lung lesion detection but not specific for malignancy, and indetermined lesions frequently require further investigation for differential diagnosis [5, 6].

2-deoxy- 2-[18 F]fluoro-D-glucose ([^18^F]-FDG) PET/CT is a key imaging modality for lung cancer staging, restaging and follow-up [7]. FDG-PET functional imaging provides information on tissue metabolism and is thus useful for characterising the indetermined lung lesions observed on CT [8].

Contemporary PET/CT scanners now implement silicon photomultiplier (SiPM) detectors in place of the conventional photomultiplier tubes (PMTs), drastically improving the time-of-flight precision and maximal count rate [9]. Additionally, present generation SiPM PET devices adopt longer axial detector extensions, compared to previous generations (~ 25–100 cm vs. ~ 15 cm) [10]. This results in improved image spatial resolution and effective sensitivity, outperforming PMT-based PET/CT scanners by improving sub-centimetre lesion detection capabilities [11, 12]. Owing to its superior diagnostic performance, different studies have confirmed the feasibility of shortening the acquisition time without compromising the image quality, which is approximately similar to reducing the injected activity [13, 14, 15]. Both are major factors for acquisition count-rate approximated by the time-activity product (TAP) [14, 16]. Reducing the acquisition time improves patient comfort, by decreasing the patient’s tendency to eventually move and enables to maximise image acquisition flow rate per PET scanner. On the other hand, effective dose remains a concern as patient radiation should be as low as reasonably achievable (ALARA principle) to keep cancer-induced risk significantly lower than the diagnostic advantage, without compromising PET result performance [17]. Achieving adequate lesion detectability with a reduction of injected activity would potentially enable a broader indication spectrum for [^18^F]-FDG PET/CT imaging such as lung cancer screening, improving its specificity by reducing the false-positive rate. As SiPM-based PET/CT systems offer new performance perspectives, we address the question of impact of reduced injected activity on lung lesion detectability by simulating different levels of total injected activity, representing an optimisation for clinical routine as well as very low-dose conditions with 10% or lower of the clinical total injected activity.

We used decimation to retrospectively reconstruct PET images with simulated reduced levels of total injected activity from the acquired full statistics PET images with typical time-activity products (TAP). Decimation stochastically discards events from the acquired PET list-mode, simulating the desired levels of injected activity depending on the frequency of discarding events. List-mode decimation simulates low-dose PET without significant bias, regarding quantitative characteristics and results in low-dose images, compared to actually acquired low-dose PET [18]. Image quality and lung lesion detection in low-dose PET is affected by increased image noise with consequent reduction of contrast-to-noise ratio (CNR) and signal-to-noise ratio (SNR) where false-positive lesions might get generated compared to the ground truth of full statistics PET dataset [19]. Drastically decreasing injected activity may affect in lesion detectability and image quality resulting in difficult readability. In the domain of artificial intelligence (AI), convolutional neural network (CNN) programs have been trained to generate full-dose images (denoised PET) from low-dose PET images. Denoising has the potential to reduce background noise while preserving or even increasing lesion contrast, restoring a low-dose PET image quality comparable to full statistics realisations. However, due to the restricted dataset contained in the low-dose PET, in which small lesions are indistinguishable from the background noise, CNN denoising may also lead to signal suppression of relevant focal uptake regions.

In this retrospective study, we investigated the impact of PET decimation and AI image denoising on lung lesion detectability at different dose levels, simulating reduced levels of injected activity.

## Material and methods

**Fig. 1:**
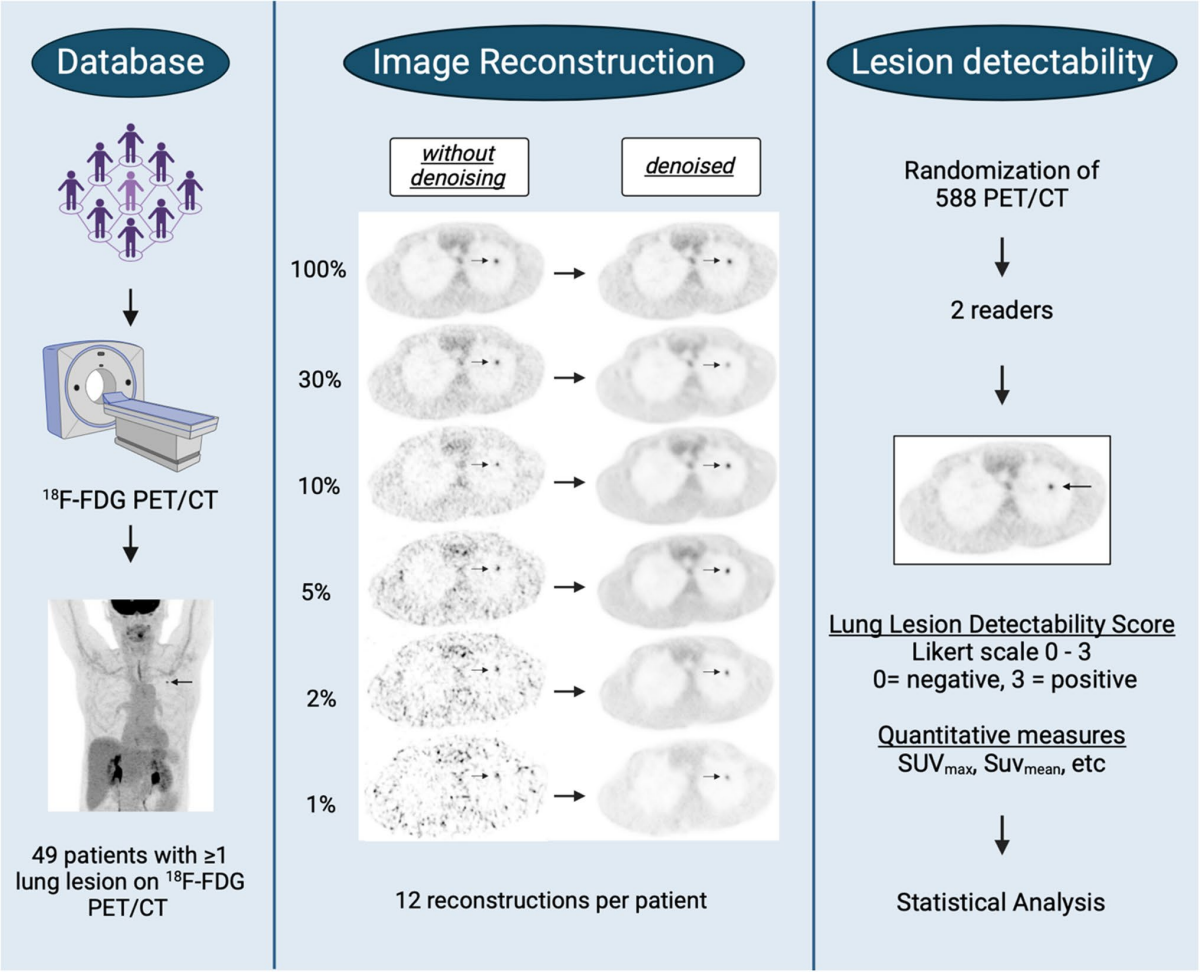
Study design of the retrospective study

The design of this retrospective study is illustrated in Fig. 1. Full statistics [^18^F]-FDG PET/CT scans were obtained in 49 patients who presented at least one lung lesion. The patients were referred for lung nodule characterisation, lung cancer staging, restaging, or follow-up. Inclusion criteria were patients over 18 years of age with a lung lesion with hypermetabolism on the acquired [^18^F]-FDG PET/CT. The exclusion criteria were minor patients or patients with lung lesions that were not [^18^F]-FDG PET avid.

The local Ethics Research Committee of the State of Vaud approved the research protocol (CER-VD #2018–01513). A general consent from CHUV was approved by all patients participating in this study, agreeing a retrospective use of their images for clinical research.

### Image acquisition

Two Biograph Vision 600 (Siemens Healthineers) with a field-of-view of 26.2 cm axial PET were used to acquire full statistics [^18^F]-FDG PET/CT images. Patients fasted for at least 6 h and had an average blood glucose of 5.9 mmol/l before administration of [^18^F]-FDG. The image acquisition followed an average of 63 min after a 2.1 MBq/kg, 2.5 MBq/kg or 3.5 MBq/kg intravenous injection of [^18^F]-FDG. 2MBq/kg is the normally injected activity in our center for [^18^F]-FDG PET, which is lower than the Diagnostic Reference Limits (DRL) of 3.5MBq/kg set in Switzerland. Some patients got higher injected activity (2.5MBq/kg or 3.5MBq/kg) because a faster patient flow was required during a certain period for which the acquisition time was proportionally adjusted. For anatomic correlation and attenuation correction, a low dose CT (100 Ref mAs; 100 kV; CARE Dose4D; CARE kV; pitch, 1; time per rotation, 0.8 s; slice thickness, 2 mm) was acquired before the whole-body continuous bed motion PET scan 1.4 mm/s (2.2 MBq/kg), 1.8 mm/s (2.5 MBq/kg) 2.2 mm/s (3.5 MBq/kg). Images were reconstructed using TrueX + TOF (UltraHD) algorithm with 3 iterations and 5 subsets with an image matrix of 440 × 440 voxels. Time-of-flight and point spread function information was used in the iterative reconstruction process, in addition to other pertinent image corrections (normalisation, dead time, activity decay, random coincidence and attenuation and scatter corrections).

### Decimation and denoising techniques

The acquired full statistics [^18^F]-FDG PET were retrospectively decimated by simulating [^18^F]-FDG PET with 30%, 10%, 5%, 2%, and 1% of the full injected activity. Low statistics PET realisations were generated for each decimation levels by parsing the original list-mode PET acquisition and randomly keeping or discarding each coincident event according to the target fraction.

Full and reduced statistics PET datasets were denoised using a CNN algorithm trained to restore PET image quality, in terms of background noise (SNR) and contrast-to-noise ratio (CNR), to the level of the original full statistics dataset. The target count levels were defined differently between the training and evaluation subject populations. Image data from 16 subjects with various tracers, that is, 4 FDG, 9 PSMA, and 3 DOTATOC, were used to train the CNN, in which input images were generated from three discrete true count levels: 5 million, 10 million, and the full statistical PET dataset respectively. After CNN training was completed, the 49 new subjects of this study were included in the evaluation dataset. The range of count levels was expanded to evaluate generalisability of AI denoising and was defined according to predefined fractions of the total number of trues: 100%, 30%, 10%, 5%, 2%, and 1%.

The 3D CNN used in this work was similar to a ResUNet architecture with 2 down-sampling steps [20]. The encoding and decoding paths were skip-connected by residual summing operations instead of channel-wise concatenation, resulting in a nested residual CNN similar to that used previously [21]. To preserve absolute quantification within the denoised network outputs, all PET inputs were scaled only by a constant factor, and normalisation operations were not used within the network itself.

The CNN was trained on input samples comprising different noise levels simultaneously. The target label was always the corresponding 100% count image, and the training objective was a combination loss, consisting of the standard mean squared error, or L2, training loss and an adversarial loss from another generative adversarial network (GAN) discriminator network. Network training and inference were performed using 3D “patches” of dimensions 64^3^, since hardware limitations made it infeasible to propagate an entire subject volume through the CNN. During training, patches were randomly selected from subject volumes to be included in the training population. During inference, the subject volume was decomposed into a set of overlapping patches, processed through the CNN, and then the denoised patches were recomposed into the space of the original subject volume. The 2% and 1% decimation levels were not included in the training samples.

All PET images were reconstructed using ordered subset expectation maximisation (OSEM) incorporating time-of-flight (TOF) and point-spread function (PSF) for three iterations and five subsets.

For each patient, we reconstructed PET datasets with 100%, 30%, 10%, 5%, 2%, and 1% of the original full statistic with and without the application of the CNN-denoising. (Fig. 1). A total of 588 (49 patients × 6 decimation levels × 2 denoised/non-denoised) PET realisations were generated for this study.

### Image analysis

The patients were separated into five groups of ten patients in which the 12 reconstructions per subject were randomly numerated and read blindly by two physicians individually (one junior without previous experience and one senior with 13 years of experience). The readers were blind to the type of reconstruction or the corresponding full statistics PET. For each PET realisation, the readers focused on lung lesions found on low-dose CT and, depending on lesion [^18^F]-FDG hypermetabolism compared to the surrounding physiological uptake, assessed the lesion detectability based on a Likert scale score (0 = negative, 1 = probably negative, 2 = probably positive, 3 = positive). In case of discordance of the two readers, a consensus was reached upon evaluation from a third senior with 25 years of experience. If the patient presented with numerous lesions, the three largest lesions per lung lobe (maximum 18 lesions per patient) were defined on CT for all reconstructions. Semi-automated segmentation of the lung lesion was proceeded on a basis of 40% of SUV_max_, and quantitative measures were reported (SUV_max_, SUV_mean_, SUV_peak_, MTV (Metabolic Tumor Volume), TLG (Total Lesion Glycolysis)) for each reconstruction. SUV_max_ and SUV_mean_ were also measured in the surrounding normal lung parenchyma to obtain the background SUV_mean_ (SUV_mean,bg_). To compensate for reduced statistics and allow quantitative comparability, in each PET realisation, corrected SUV measurements were obtained multiplying the measured SUV for the corresponding scaling factor (SF) of 100, 50, 20, 10, 3.33 and 1 for the 1%, 2%, 5%, 10%, 30%, and 100% decimation levels, respectively:

$${SUV}_{corrected}={SUV}_{measured}\times SF$$

For each lung lesion and each PET realisation, the ratio of lesion SUV_max_ to SUV_mean,bg_ was defined as rSUV:

$$rSUV=\frac{{SUV}_{max} }{{SUV}_{mean,bg}}$$

### Statistical analysis

Lung lesion detectability and quantitative measurements between the full statistics and decimated datasets were separated into two groups: denoised and non-denoised datasets. The detectability score was divided into positive (1 if Likert score = 2 or 3, probably positive or positive) and negative (0 if Likert score = 0 or 1, probably negative or negative) malignancy scores. The detectability score was separated into a certain score (1 if Likert score = 0 or 3) and an uncertain score (0 if Likert score = 1 or 2). Pearson chi-squared test of homogeneity was performed on detectability score (0, 3) and dichotomised malignancy score (0, 1) between decimations on both groups. The test of homogeneity was also calculated at the same decimation levels by comparing the detectability and malignancy score distributions between denoised and non-denoised PET. The same statistical tests were performed on lesions smaller than 4 mm (< 4 mm) and lesions corresponding to the lung nodule size criteria (4–30 mm). ANOVA was used on SUV_max_ and rSUV between decimation levels on denoised and non-denoised reconstructions, respectively. SUV_max_ and SUV_mean_ were analyzed in relation to detectability scores across different decimation levels, both with and without denoising. Subgroup analyses were also performed to assess the impact of lesion multiplicity and anatomical location on detectability. Lung lesions were stratified into (a) upper versus middle/lower lobes and (b) patients with a single lesion versus those with multiple lesions. Detectability scores were compared at each decimation level using Pearson chi-squared tests, separately for denoised and non-denoised datasets. All results are presented in Supplementary Table S2.

## Results

### Study population

We performed [^18^F]-FDG PET/CT in 49 patients (57% (*n* = 28) male and 43% (*n* = 21) female, aged 65 ± 10 years, and BMI 24.3 ± 5.8 kg/m^2^) acquired between February and November 2023 at the Lausanne University Hospital (CHUV). Patient characteristics are listed in Table 1. The indications for PET imaging were lung nodule characterisation in 45% (*n* = 22), lung cancer staging in 12% (*n* = 6), re-staging in 33% (*n* = 16), and follow-up in 10% (*n* = 5) of the patients. Of the 49 patients, the following diagnoses were confirmed by lung biopsy: 45% (*n* = 22) had non-small cell lung cancer (NSCLC), 6% (*n* = 3) had small cell lung cancer (SCLC), 4% (*n* = 2) had pulmonary carcinoid, 8% (*n* = 4) had lung metastasis, 12% (*n* = 6) had no malignancy, and 2% (*n* = 1) had no conclusive biopsy. No histology was observed in 24% (*n* = 12) of the patients, and 43% (*n* = 21) were already known for other types of cancer. Tobacco use was reported in 73% (*n* = 36) of patients, including 37% (*n* = 18) with COPD, 10% (*n* = 5) were non-smokers, and the rest were not reported.

**Table 1:** Patient characteristics

**Patient characteristics**			
Patients (*n* = 49)		**PET Indication**	
Male	57%	characterisation	45%
Female	43%	follow-up	10%
Age	65.1 ± 10.4	staging	12%
BMI	24.3 ± 5.8	re-staging	33%
Glycemia before injection	5.9 ± 1.2 mmol/l		
Lung histology		**Injected Activity**	
NSCLC	45%	2. MBq/kg	41%
SCLC	6%	2.5MBq/kg	10%
carcinoid tumour	4%	3.5MBq/kg	49%
Metastasis	8%		
No malignity	10%		
Biopsy not conclusive	2%		
no histology of lung	24%		
Other known cancer	43%		

### SUV, rSUV, MTV and TLG analysis

We analyzed 141 lung lesions with a diameter of 12 mm (standard deviation: ± 12 mm [range 4–30 mm]) across 588 (12 × 49) reconstructions resulting a total of 1692 observations. Across all decimations, the mean lesion SUV_max_ were 8.3 ± 9.9 g/mL and 4.7 ± 5.9 g/mL, the mean rSUV on contoured lesions were 14.7 ± 26.2 and 14.1 ± 22.1 for non-denoised and denoised datasets respectively. At the same count level, SUV_max_ values were significantly different when comparing reconstructions without and with denoising for 10%, 5%, 2%, and 1% decimation (*p* < 0.017) and rSUV at 30% decimation level only (*p* < 0.001). Compared to the corresponding full statistics, the SUV_max_ differed significantly at 2% and 1% decimation levels without denoising and the rSUV at 2% and 1% without denoising and at 1% decimation level with denoising. MTV was 0.8 ± 2.6 mL without denoising and 1.7 ± 4.7 mL with denoising and for TLG 2.0 ± 19.3 g and 2.8 ± 24.9 g. Supplementary table S1 lists all mean values of SUV_max_, SUV_mean_, SUV_peak_, rSUV, MTV, TLG, SUV_max,bg_ and SUV_mean,bg_ at each count levels with and without denoising including the p-values of the pairwise mean comparisons between different decimation levels with and without denoising. SUV_max_ and SUV_mean_ of lung lesions were calculated per decimation with and without denoising for each detectability score. The results are listed in Supplementary Table S2.

### Detectability analysis

**Fig. 2:**
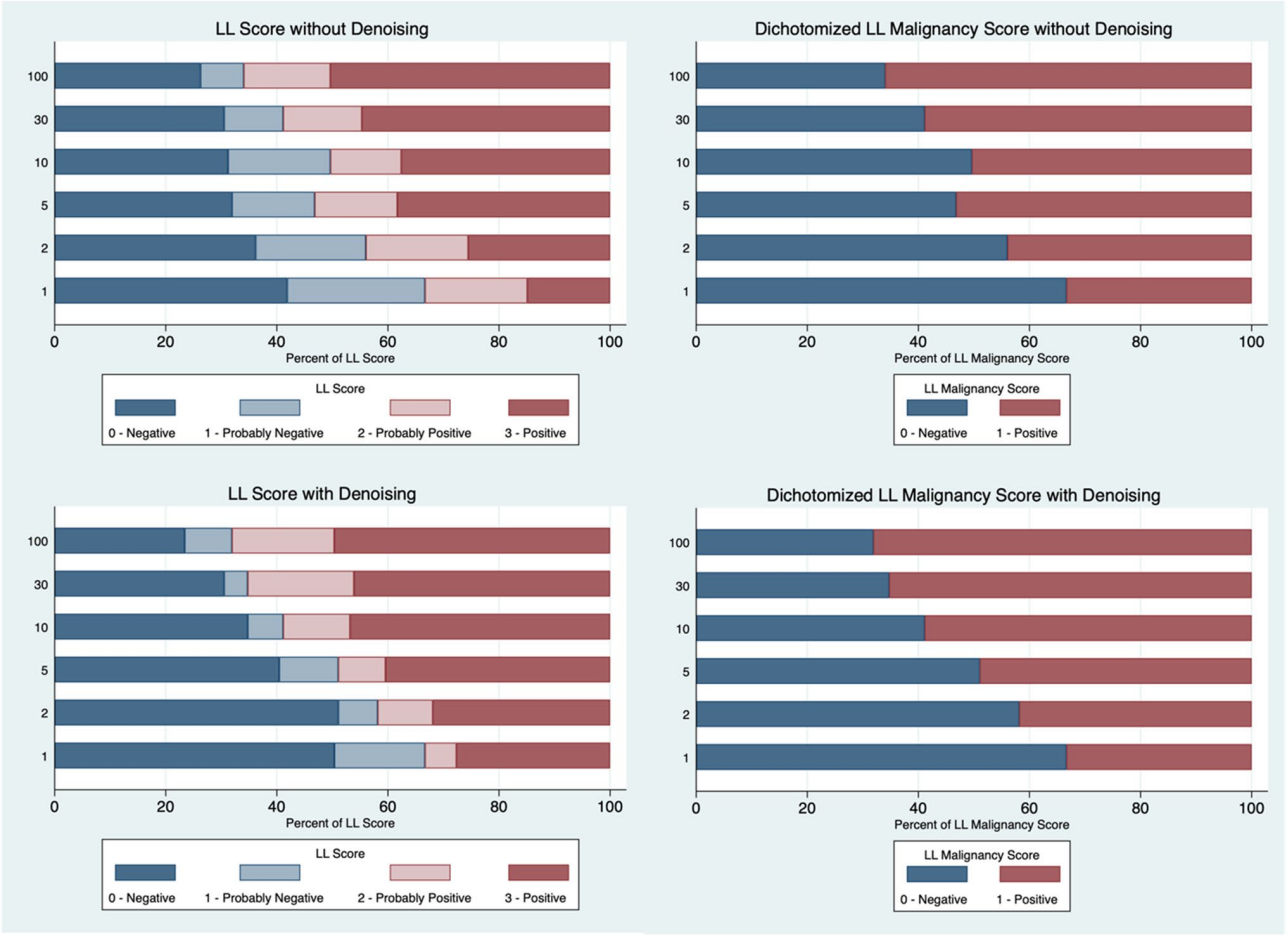
Distribution of lung lesion (LL) detectability score (left column) and lung lesion dichotomised malignancy score (right column) for each decimation level with and without denoising

The final lung lesion detectability score and dichotomised malignancy score distribution for each decimation level are shown in Fig. 2. Full statistics PET contained 66% positive and 34% negative malignancy scores, while denoising slightly increased the positive fraction for the malignancy score (68.1% and 31.9%, respectively). Reducing the simulated injected activity to 1%, the lung malignancy score distribution was reversed, with 33.3% positive and 66.7% negative with and without denoising.

Compared to full statistics, lung lesion (LL) Likert detectability score distribution differed significantly at 2% and 1% decimation without denoising (*p* < 0.001) and at 5%, 2%, and 1% with denoising (*p* < 0.001). The dichotomised malignancy score was significantly different at 10%, 5%, 2%, and 1% without denoising (*p* < 0.029) and at 5%, 2%, and 1% with denoising (*p* < 0.001). Results were similar for the subgroup analysis of lesions corresponding to lung nodule size criteria only (*p* < 0.009). There was no significant difference in the score distribution between the lung lesions smaller than 4 mm. Comparing homogeneity of score distribution between denoised and non-denoised reconstructions at the same count levels, the lung lesion detectability score was significantly different at 10%, 2%, and 1% (*p* < 0.019), while there was no significant difference in the malignancy score. Denoising increased the lung lesion score certitude given by readers and significantly reduced the proportion of scores 2 (probably positive) and 1 (probably negative) from 10% decimations and lower (*p* < 0.038). The use of denoising led to a notable reduction in the proportion of lesions assigned an uncertain score, defined as Likert 1 or 2 (‘probably negative’ or ‘probably positive’). At 100% injected activity, 23% of lesions fell into this uncertain category, and this proportion increased progressively to 43% at 1% decimation without denoising. In contrast, when denoising was applied, the proportion of uncertain scores remained relatively stable across all decimation levels.

In the upper and lower lobe subgroups, detectability scores were compared to full-statistics PET at each decimation level. Without denoising, significant differences from full statistics were observed at 2% (*p* = 0.001 upper, *p* = 0.024 lower) and 1% (*p* < 0.001 upper, *p* = 0.002 lower). With denoising, detectability differed significantly at 2% (*p* = 0.049 upper) and 1% (*p* < 0.001 upper), while lower lobe scores at 1% did not differ significantly (*p* = 0.404).

In the single versus multiple lesion subgroup analysis, significant detectability differences were found at 2% (*p* = 0.017) and 1% (*p* < 0.001) for single-lesion patients without denoising, and at 5% (*p* = 0.045), 2% (p < 0.001), and 1% (*p* = 0.002) with denoising for multiple-lesion patients.

## Discussion

In this retrospective study, we evaluated the effect of [^18^F]-FDG PET/CT decimation on lung lesion detectability. By simulating reduced injected activity, the lung lesion dichotomised malignancy score was preserved up to 30% for PET reconstructions without denoising and up to 10% for denoised PET. The lung lesion score distribution was significantly different for non-denoised PET from 5% decimation but from 10% for denoised PET reconstructions. We explain the difference in performance of denoising on dichotomised malignancy score compared to lung lesion score as the lung lesion detectability score continuously degrades from positive [3] to eventually negative (0) with lower decimation without denoising. Because denoising enhances contrast and reduces noise, denoised decimation tends to change the lung lesion score drastically between count levels, changing the lung lesion score distribution significantly at higher injected activity than without denoising. Nevertheless, denoising was more effective for dichotomised malignancy score detectability as it significantly increased the fraction of positive lesions at 10% decimation and enhanced the certitude of readers’ judgement from 10% decimation and below. Denoising significantly reduced the proportion of lesions scored as ‘probably positive’ or ‘probably negative,’ increasing diagnostic confidence—a clinically relevant shift not fully reflected in dichotomized metrics. Compared to the full statistics, SUV_max_ means analysis differed significantly on very low statistics (2% and 1%) without denoising underlying the important background noise levels, resulting in an extreme range of SUV_max_ values that were smoothed by denoising. Across decimation levels, SUV_max_ values were more stable than SUV_mean_, particularly with denoising. This suggests that SUV_max_ is more robust for lesion detectability in low-dose settings, while SUV_mean_ is more affected by noise and segmentation uncertainty, limiting its reliability for lesion characterization. These observations are supported by the subgroup data in Supplementary Table S2.

To assess lung lesion detectability, decimation enabled an accurate simulation of PET/CT images acquired with different injected activities, without the need for further image acquisition. Without denoising, decimation showed adequate image quality, particularly at 30% of the injected activity, where its reconstruction could be mistaken for full statistics. Even though the lung lesion malignancy score was significantly lower at 10%, the general image quality was maintained with low noise on the lung parenchyma and clear structure delimitations. Detectability was continuously degraded by reducing the number of counts.

**Fig. 3:**
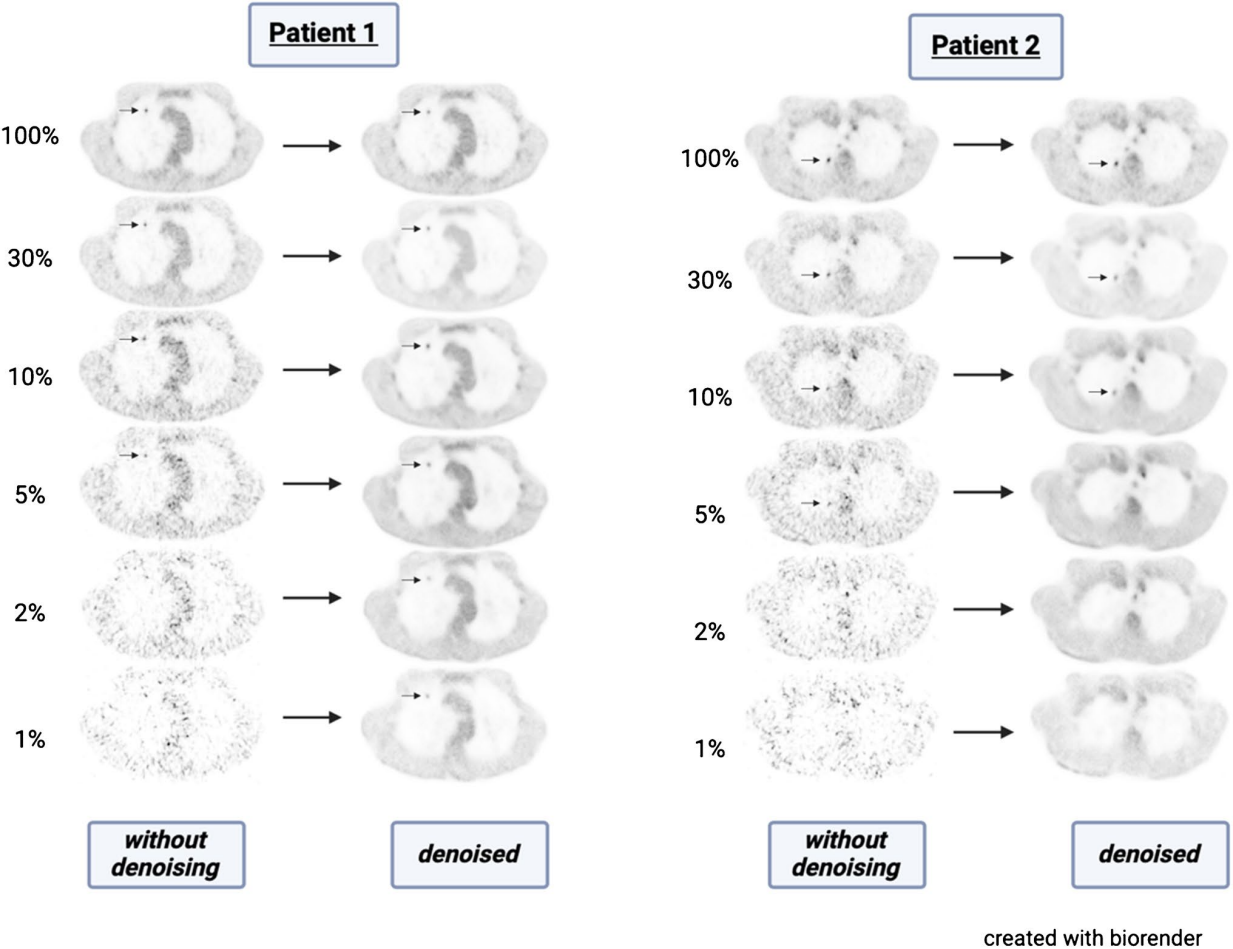
Example of 5 decimation levels of [^18^F]-FDG PET with and without denoising in two patients. The lung lesion score stays positive for all reconstructions on patient 1. On patient 2 the lung lesion detectability score becomes negative in the 2% and 1% decimations

We found that denoising is essential for drastically improving the readability of PET with reduced statistics, especially for extremely low-dose PET. Homogenised physiological uptake and enhanced lesion contours accelerated reading and increased the certitude for lung lesion evaluation. Although spreading noise into a homogeneous background improves the image quality, the adverse effect of erasing the remaining counts of a small lung lesion remains a challenge. The last effect was observed on some small lesions at very low-statistics presenting discrete hypermetabolism at full statistics, where denoising on low decimation would consider the remaining [^18^F]-FDG signal as noise and spread it into a homogeneous background, resulting in the complete disappearance of the lesion, as shown in Fig. 3. Thus, denoising can suppress focal uptake in small lesions at very low count levels by interpreting them as background noise, potentially leading to false negatives. This risk must be carefully evaluated before considering such very low count levels in clinical application. Nevertheless, denoising has not been specifically trained on [^18^F]-FDG radiotracer for PET/CT, and the presence of lung lesions was not an inclusion criterion for the dataset as the denoising was trained for whole-body denoising. In this study, we focused on evaluating the effect of denoising on the lung lesions. Denoising could thus be improved for very low-dose lung lesion screening if it is specifically trained on [^18^F]-FDG PET/CT with a subsample of patients presenting with various benign and malignant lung lesions to improve its accuracy and generalizability. Across all reconstructions, we did not observe any appearance of false-positive lesions on decimated PET/CT.

The decimation method essentially creates a worst-case scenario by using low true counts and high random fractions. Even though it scales the prompts and scatter events correctly, the fraction of random events is artificially high for low-count realisations, as it scales quadratically with the in-field activity but remains consistent with that of the full statistics scan*.* However, a clinical validation of the decimation method was published by Schaefferkoetter et al. [18] and demonstrated robustness of the randoms correction and accuracy of the PET quantification in the reconstructed images. Acquired low-dose [^18^F]-FDG PET/CT were compared to decimated full statistics [^18^F]-FDG PET/CT to the same number of counts as the acquired low-dose acquisition. No quantitative bias from low-dose PET emulation was observed, supporting the methodology used in our retrospective study. The under-representation of true counts is an advantage of our study, which further supports the feasibility of reducing injected activity for lung lesion detectability.

Schaefferkoetter et al. [22] investigated the lung lesion detectability of low-dose [^18^F]-FDG PET/CT in 12 lesions regarding the possibility of lung cancer screening by taking full dose PET and decimated PET. Their results showed that the injected activity could be reduced to 10 million true counts while maintaining lung lesion detectability. This study assessed a smaller scale of lung lesions, and PET/CT images were acquired on a Biograph mCT, preventing us from comparing the number of true counts to our results. However, their results support the observed preservation of lung lesion detectability on PET/CT with reduced injected activity. To the best of our knowledge, lung lesion detectability has not been yet studied using decimated and denoised [^18^F]-FDG PET/CT in many lesions. This observer-based study provides information supporting that lesion detectability may be preserved at reduced activity levels. These results advocate the design of future prospective studies rather than immediate adoption for screening. Further studies with larger datasets of small nodules including patients without lung lesions should be assessed to deepen applicability of low-dose ^18^F-FDG PET/CT in lung cancer screening. As low-dose CT remains the current reference modality for lung cancer screening, evaluating the added value of reduced-activity [^18^F]-FDG PET/CT requires comparative analysis. This study was conceived as a technical feasibility assessment focusing on lesion detectability at reduced activity levels, rather than as a direct clinical comparison with low-dose CT. Although a comparative evaluation was beyond the scope of this work, further prospective studies—including in asymptomatic populations—are warranted to determine whether low-dose PET/CT could complement or enhance existing screening strategies.

Reducing the injected activity is of great interest in paediatric patients, as children are more sensitive to radiation-induced cancer. Zhao et al. [23] investigated image quality and lesion detectability in paediatrics oncological [^18^F]-FDG PET/CT by retrospectively simulating reduced time acquisitions. They defined a reduction of 1/10 of the injected dose as the best compromise in terms of image quality and microlesion detectability, with the limiting factor being first poor image quality before losing microlesion detection. As observed in our study, the image quality of low statistics can be drastically improved by denoising, and further reduction of the injected activity is potentially conceivable.

New promising tracers are emerging, such as [^68^Ga]-FAPI- 46 PET, a potential tracer for the differential diagnosis of lung lesions that are negative on [^18^F]-FDG PET/CT. Röhrich et al. [24] observed different patterns in tracer-uptake by taking dynamic and static ^68^Ga-FAPI- 46 PET/CT of lung lesion negative on [^18^F]-FDG PET/CT, which could discriminate benign from malignant lesions. The latter study shows promising new perspectives for achieving a differential diagnosis without invasive biopsy. Investigating the potential of low-dose [^68^Ga]-FAPI- 46 PET/CT could be of interest, as the latter presents different properties in specificity and could potentially be introduced in lung lesion initial evaluation.

Different methods for denoising PET/CT have emerged as for example commercially available SubtlePET enabling to reduce injected activity without comprising image quality [25]. In our study, we tested one method but in future perspective it would be of interest to compare the performance of different approaches. Artificial Intelligence has also been trained for the automatic detection of lung lesions on different full statistics and low-dose [^18^F]-FDG PET/CT by Schwyzer et al. [26] and showed performant detectability along with a reduction in the injected activity. Automatic detection could be useful as support for physicians in lung cancer screening.

This retrospective study has several limitations. It was pursued in one centre using two Siemens Vision 600 Biographs, which did not include common variations in image acquisition between different PET/CT scanners. External validation using data from other centers and scanner platforms is necessary to confirm the generalizability of these findings. The image acquisition protocol had different injected activities, as the centre had to increase the number of acquisitions per PET scanner for a certain period; nevertheless, the acquisition time was proportionally adapted, and the full statistics maximal count rate is expected to be similar. Another limitation was our database, which contained lung lesions of various sizes and characteristics. Despite randomization and blinding, the repeated reading of 12 reconstructions per patient could have introduced a learning bias, as lesion patterns may have been recognized across reconstructions. Although lesion scoring was based on comparison to physiological uptake rather than memory, this limitation is acknowledged.

While denoising improved image quality and reader confidence, it may also have some limitations. The algorithm can smooth out the background, which helps reduce noise but might also hide small lesions, especially at very low activity levels. In some cases, this could lead to underestimation or even disappearance of true lesions. Future studies should further explore these risks to better understand the effects of AI-based denoising in clinical PET imaging. Our analysis specifically examined denoising effects on pulmonary lesions, although the CNN model was originally developed for general whole-body PET denoising rather than task-specific training. This lack of task-specific optimization could reduce its robustness for small lesion enhancement at ultra-low statistics. Tailored training on lung-focused [^18^F]-FDG datasets may enhance future performance.

Despite these limitations, we analysed the detectability of 141 lung lesions from 49 patients across 588 PET/CT scans on low-dose and denoised PET/CT without requiring further image acquisition. Besides the variety in lung lesion characteristics, the results from the lung lesion subgroup of lung nodule size only were similar. Our results are robust, as the used decimation tool is based on a worst-case scenario that prevents overestimation of the diagnostic potential and image quality of low-dose PET/CT. This observer-based study provides feasibility data suggesting that lesion detectability may be preserved at reduced activity levels. These results support the design of future prospective studies rather than immediate adoption for screening. Further studies with larger datasets of small nodules including patients without lung lesions should be assessed to deepen applicability of low-dose [^18^F]-FDG PET/CT in lung cancer screening.

## Conclusion

In this retrospective study, we analysed the impact of PET decimation and AI image denoising on lung lesion detectability simulating different levels of injected activity representing activity optimisation in routine clinical practice as well as very low-dose conditions for lung cancer screening. According to our results, injected activity could be reduced to 30% without denoising and 10% with denoising, while still maintaining lung lesion detectability. Image quality, readability, lung lesion detectability and diagnostic confidence were increased by denoising. These findings support the technical feasibility of reduced-activity PET/CT for lung lesion assessment. However, further validation—including comparisons with low-dose CT and prospective studies in asymptomatic populations—is essential to clarify its potential role in lung cancer screening.

## Supplementary Information

Below is the link to the electronic supplementary material.

Supplementary file1 (XLSX 15 KB)

Supplementary file2 (XLSX 15 KB)

## Data Availability

The results and datasets obtained during this retrospective study are available from the corresponding authors on reasonable request.
